# Modulation of Determinant Factors to Improve Therapeutic Combinations with Immune Checkpoint Inhibitors

**DOI:** 10.3390/cells9071727

**Published:** 2020-07-19

**Authors:** Magalie Dosset, Elodie Lauret-Marie Joseph, Thaiz Rivera Vargas, Lionel Apetoh

**Affiliations:** 1 The Laboratory of Immunology, Department of Medicine and Moores Cancer Center, University of California, San Diego, 9500 Gilman Drive, La Jolla, CA 92093-0815, USA; magalie.dosset@gmail.com; 2 Ludwig Institute for Cancer Research, University of Lausanne, Agora Center, Rue du Bugnon 25A, 1005 Lausanne, Switzerland; elodie.lauretmj@gmail.com; 3 INSERM, U1231, 21000 Dijon, France; rivera.thaiz@gmail.com; 4 Faculté de Médecine, Université de Bourgogne Franche Comté, 21000 Dijon, France

**Keywords:** immunogenic therapy, immune checkpoint inhibitors, combined therapies, cancer, T cells, stem-cell like memory T cells, resident-memory T cells, vaccine, chemotherapy, radiotherapy

## Abstract

Immune checkpoint inhibitors (ICPi) have shown their superiority over conventional therapies to treat some cancers. ICPi are effective against immunogenic tumors. However, patients with tumors poorly infiltrated with immune cells do not respond to ICPi. Combining ICPi with other anticancer therapies such as chemotherapy, radiation, or vaccines, which can stimulate the immune system and recruit antitumor T cells into the tumor bed, may be a relevant strategy to increase the proportion of responding patients. Such an approach still raises the following questions: What are the immunological features modulated by immunogenic therapies that can be critical to ensure not only immediate but also long-lasting tumor protection? How must the combined treatments be administered to the patients to harness their full potential while limiting adverse immunological events? Here, we address these points by reviewing how immunogenic anticancer therapies can provide novel therapeutic opportunities upon combination with ICPi. We discuss their ability to create a permissive tumor microenvironment through the generation of inflamed tumors and stimulation of memory T cells such as resident (T_RM_) and stem-cell like (T_SCM_) cells. We eventually underscore the importance of sequence, dose, and duration of the combined anticancer therapies to design optimal and successful cancer immunotherapy strategies.

## 1. Introduction

It is now well-established that the emergence and propagation of tumor cells are initially controlled by the immune system of the host [1]. However, cancer cells gradually develop several immunosuppressive mechanisms that can ultimately overwhelm the natural defense of the host and lead to cancer spreading. Among the different subsets of immune cells, T cells that specifically recognize tumor antigen-expressing cells act as key orchestrators and effectors of the antitumor immune response [2,3]. In particular, CD4 T_H_1 cells characterized by the secretion of IFNγ (Interferon gamma)-associated cytokines can not only contribute to direct tumor cell killing but also endow CD8 T and NK (natural killer) cells with optimal cytotoxic functions [4,5,6]. The benefit of a T_H_1-associated immune signature has been demonstrated in several cancers [7,8]. However, this strong antitumor immunity is accompanied by the gradual occurrence of inhibitory mechanisms that will hamper the activity of immune cells and turn off their functions [9,10,11,12]. The accumulation of immunosuppressive cells such as Regulatory T cells (Tregs) and Myeloid-derived Suppressor Cells (MDSC) can compromise anticancer immune responses [11]. Likewise, the cell-surface expression of inhibitory molecules on activated T cells contributes to a progressive inhibition of the immune response [10,13,14]. This underscores the challenge for cancer therapeutics to initiate a long-lasting effective antitumor T cell immunity.

Anticancer therapies can be referred to as immunogenic when they induce an immune response. This encapsulates therapies that are able to deplete immunosuppressive cells or promote T cell activation. Since the 1940s, chemotherapy was the main option to treat advanced cancer because of its direct cytotoxicity on tumor cells. The immunogenic properties of some cytotoxic chemotherapies were subsequently characterized [15,16]. For example, 5-Fluorouracil (5-FU) and Gemcitabine deplete myeloid suppressive cells, thereby restoring the ability of T cells to enter the tumor and secrete cytokines [17,18]. Platinum-based chemotherapies such as oxaliplatin can induce an immunogenic form of tumor cell death (ICD) by promoting the cell surface expression of calreticulin (CRT) and the release of danger signals such as ATP (adenosine triphosphate) and HMGB1 (High mobility group box 1 protein), which are detected by immune cells [19,20]. Radiation therapy was also shown to mobilize antitumor immunity [21,22]. However, in clinical settings, mono- or poly-conventional therapies often fail to achieve complete cancer cure and long-term survival.

Vaccines are another type of immunogenic anticancer therapy, which relies on immunizing patients against tumor antigens and induces a specific effector and memory T cell immunity against tumor cells. Therapeutic vaccines have been tested in patients refractory to conventional therapies such as surgery, chemotherapy, or radiotherapy. For decades, many efforts were invested in the development of therapeutic cancer vaccines. Unfortunately, their efficiency in animal models as single therapy has not been translated to humans. Although Sipuleucel-T (Provenge), an antigen-presenting cell-based immunotherapy for castration-resistant metastatic prostate cancer, was initially approved by the U.S Food and Drug Administration (FDA), its efficacy remains limited.

Cancer treatment with immune checkpoint inhibitors (ICPi) was a milestone in cancer therapy and progressively became a standard of care to treat several human cancers. ICPi aim to promote T cell reactivation or prevent their dysfunction by the use of blocking monoclonal antibodies targeting immunosuppressive molecules such as CTLA-4 (cytotoxic T-lymphocyte-associated protein 4), PD-1 (Programmed cell death 1), and TIM-3 (T-cell immunoglobulin mucin-3), which are called immune checkpoints. Despite this significant progress, a substantial number of patients are unresponsive to ICPi therapy from the very start [23], while others progressively develop a resistance to treatment [24]. Furthermore, certain cancers are also more refractory than others. For instance, 80% of colorectal cancers are unresponsive to ICPi therapy [25], while the figures are 50–70% for lung cancers [26] and 20–30% for melanomas [27]. Tumor analysis revealed that the response to ICPi often correlates with the pre-existence of an immunologically privileged tumor microenvironment characterized by the presence of CD4/CD8 T cells, immune checkpoints, and pro-inflammatory cytokines [28,29]. Thus, a therapeutic pre-stimulation of T cell immunity may be the key to unleash the full potential of ICPi therapy [30].

In this review, we discuss the potential of immunogenic anticancer therapies to modulate critical immune factors associated to the response to ICPi therapy. We will notably focus on memory stem-cell like (T_SCM_) and tissue resident memory T cells (T_RM_), which are two recently characterized effector T subsets that support the efficacy of ICPi therapy. Finally, we discuss the importance of treatment sequence, dose, and duration in the design of immunologically relevant, optimized and safe therapeutic combinations.

## 2. Immunological Parameters Accounting for Improved Efficacy of Combined Immunotherapies

### 2.1. Immunogenic Anticancer Therapies Convert ‘Cold’ Tumors into ‘Hot’ Tumors

Upon antigen exposure, activated T cells progressively upregulate the cell surface expression of ICP to prevent any uncontrolled reactivity that could damage healthy tissues. T cell dysfunction refers to an altered functionality of ICP-expressing T cells resulting from persistent antigen exposure and triggering of ICP by their ligands [10,11,31]. In cancer, ICPi have been intensively investigated as targets, hoping to restore/prevent the dysfunction of antitumor T cells in the tumor microenvironment. The pre-existence of intratumor T cells, ICPi, and IFNγ-related immune signature accounts for an ‘inflamed’ tumor microenvironment, which is usually associated with a positive response to ICPi therapy [32]. This suggests that the presence of functional tumor-specific ICP^+^ T cells may be a favorable predictive marker for the success of ICPi therapy. Hence, it is likely that using therapeutic strategies fostering the accumulation of non-dysfunctional IFNγ-secreting T cells within the tumor could dramatically improve the efficacy of ICPi therapy.

**Figure 1:**
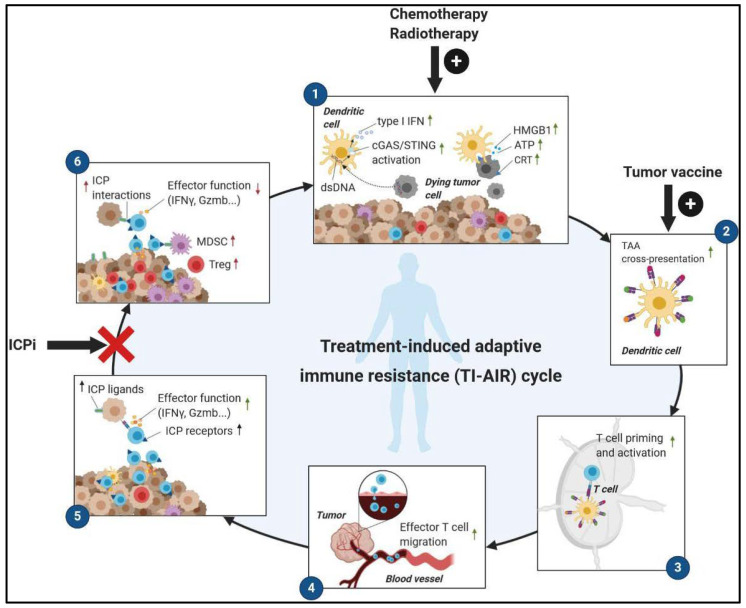
Treatment-induced adaptive immune resistance (TI-AIR) with immune checkpoint inhibitors (ICPi). The efficacy of immunogenic anticancer therapies fits into a cyclic process that starts with steps leading to the stimulation of an antitumor T cell response and ends with the occurrence of a negative immunomodulatory feedback loop that curtails T cell expansion and effector functions, leading to tumor outgrowth. However, the disruption of this treatment-induced adaptive immune resistance can be achieved by the combination of immunogenic therapies with immune checkpoint inhibitors (ICPi) that prevent T cell dysfunction mediated by ICP interactions expressed on activated T cells and other immune/tumor cells. CRT, calreticulin; HMGB1, high–mobility group box 1; ATP, adenosine triphosphate; cGAS, cyclic GMP–AMP synthase; STING, stimulator of interferon genes; TAA, tumor-associated antigen; ICP, immune checkpoint.

In mouse models, immunogenic therapies that are associated with ICD induction and the depletion of immunosuppressive cells seem to be particularly efficient for enhancing the effect of the PD-1/PD-L1 blockade [33,34,35]. Our group reported in two mouse models of colorectal cancer that high levels of IFNγ secretion, PD-L1 expression, and PD-1^+^ memory CD8 T cells in the tumor were induced upon Folfox (5-FU plus Oxaliplatin) immunogenic chemotherapy treatment [34], leading to the conversion of ‘cold’ tumor into ‘hot’ tumors [28]. IFNγ secreted by early dysfunctional T cells was responsible for the progressive expression of PD-L1 induced on tumor cells, which in turn became able to convert effector CD8 T cells into dysfunctional cells. This negative feedback loop represents a state of treatment-induced adaptive immune resistance (TI-AIR) that promotes tumor outgrowth [36] (Figure 1). However, this TI-AIR mechanism can be disrupted by the co-administration of anti-PD-1 blocking antibodies to prevent the dysfunction of specific ICP^+^ CD8 T cell induced by Folfox, thus resulting in complete tumor regressions in most mice [34]. A similar mechanism and antitumor benefit were described in tumor-bearing mice treated with radiotherapy plus ICPi [37,38,39]. Indeed, localized radiotherapy can enhance tumor antigenicity, adjuvanticity, and immunogenicity through the increase of MHC (Major Histocompatibility Complex) class-I expression, the induction of cGas (cyclic GMP–AMP synthase)/ STING (stimulator of interferon genes)/ IFN-I (type-I interferon) pathways, and the induction of ICD. This results in massive T cell priming and recruitment into local but also distant tumors (abscopal effect) [40]. The association of cancer vaccines with ICPi also conferred a similar antitumor benefit compared to ICPi alone. In mouse models, a GM-CSF (Granulocyte-macrophage colony-stimulating factor)-secreting cancer vaccine (GVAX) improved the survival of mice engrafted with colon and pancreatic cancer cells when combined with the PD-1 blockade [41,42]. The same observation was made in multiple mouse tumor models for cyclic dinucleotides formulated GVAX (termed “STINGVAX”)/anti-PD-1 combination [43] or other cancer vaccines combined with ICPi [44,45,46].

In colorectal cancer patients, we observed a similar induction of CD8 T cells and ICP after Folfox chemotherapy [34], suggesting that the above immune modulation observed in mice might translate in humans. Importantly, the benefits of Folfox in combination with anti-PD-1 antibody (Pembrolizumab) were studied in a phase I clinical trial conducted in colorectal cancer (CRC). Most patients (27 out of 30) had a microsatellite stable (MSS)-CRC, which is a type of cancer classified as ‘cold’ tumor and refractory to anti-PD-1 blockade. Folfox/anti-PD-1 treatment resulted in 16 patients (53%) with partial/complete response and 14 patients (47%) with stable disease [47]. In another study conducted in multiple human cancers, the addition of anti-PD-L1 tends to increase the antitumor efficacy of a Folfox-based therapy, but longer follow-up would be required to confirm the immune and clinical benefit of this combined therapy [48]. A study investigating the survival benefit of Folfox combined to anti-PD-L1/anti-CTLA-4 is also currently ongoing [49]. Likewise, the ability of ICPi to improve the effect chemotherapy was observed in melanoma patients, doubling the median progression-free survival (PFS) compared to the group previously untreated by chemotherapy [50]. The tandem anti-PD-1/radiation in non-small cell lung cancer led to comparable result [51]. In a phase II clinical trial on advanced non-small cell lung cancer (NSCLC), 55% of subjects (33 out of 60) who received anti-PD-1 therapy plus a platinum-based drug achieved an objective response compared to 29% (18 out of 63) in the arm treated with chemotherapy alone [52]. The benefit of similar combination therapy in lung cancer was confirmed in a meta-analysis including 8 clinical trials and more than 4000 patients. Overall, a clear overall survival (OS) and PFS advantage was observed in the platinum-based chemotherapy plus anti-PD-1 or anti-PD-L1 groups compared to chemotherapy alone, irrespective of tumor PD-L1 level [53]. However, the degree of tumor PD-L1 expression still seemed to affect the OS, because a high PD-L1 score at baseline tends to improve the antitumor efficacy of the combined treatment [54,55,56]. Therefore, this indicates that tumor PD-L1 expression positively affects the response to anti-PD-1/PD-L1 therapy and that chemotherapy may drive PD-L1 induction, at least in tumors initially determined as PD-L1 negative. Radiotherapy combined with anti-CTLA-4 provides significant clinical benefit as well [57]. Of note, the extent of immune stimulation driven by conventional therapies can also be affected by their direct cytotoxic activity on cancer cells, which means that patients with treatment-insensitive tumors may not benefit from their combination with ICPi. This is underscored by observations that in lung cancer patients treated by platinum-based chemotherapy, the benefit of having a pre-existing antitumor T cell immunity before treatment was only notable in patients with a controlled tumor burden. Thus, the induction, at least to some extent, of tumor cell death is required for the immunostimulatory properties of chemotherapy [58,59].

Finally, the use of a human papillomavirus (HPV)-based vaccine in HPV16-positive cancer patients was reported to considerably amplify the efficacy of anti-PD-1 therapy [60]. Another study also observed an increase of PD-L1 expression after two weeks in tumors of pancreatic cancer patients treated with GVAX/Cyclophosphamide versus untreated patients [42]. However, whether this induction is attributable to the GVAX vaccine or to the chemotherapy injected remains unclear. The therapeutic efficacy of the GVAX/Cyclophosphamide and anti-PD-1 combination is currently evaluated in a clinical trial.

### 2.2. Boosting the Generation of Memory T Cells with Superior Antitumor Properties

**Figure 2:**
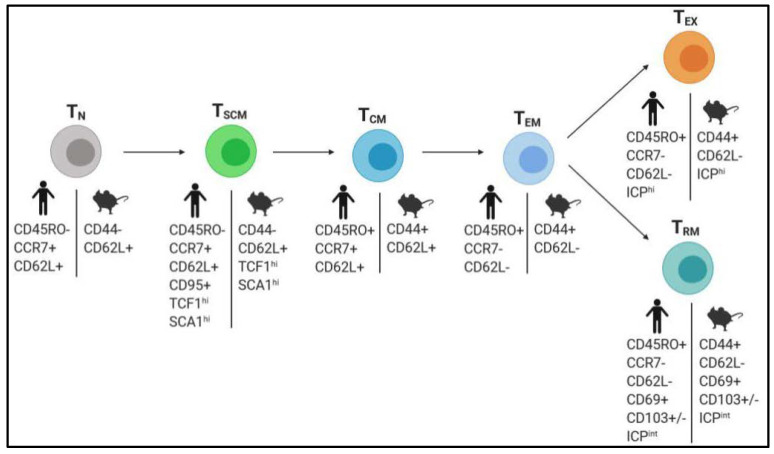
Core phenotypic characterization of memory T cell subsets. T_N_, naïve T cell; T_SCM_, stem cell-like memory T cells; T_CM_: central memory T cell; T_EM_, effector memory T cell; T_RM_, resident-memory T cells; T_EX_, exhausted/dysfunctional T cell; ICP, immune checkpoint.

Memory T cells (T_CM_) (a bystander subset generated after a primary infection and located in secondary lymphoid organs) and effector memory T cells (T_EM_) (that circulate through tissues and display antitumor functions) have been intensively studied over the past years. However, two novel subpopulations of memory T cells with superior features were recently identified as ‘stem’ cell-like memory T cells (T_SCM_) and resident-memory T cells (T_RM_) (Figure 2).

#### 2.2.1. Stimulation of T_SCM_ Cells

Stem cell-like memory T cells (T_SCM_) represent a rare population of long-lived self-renewing multipotent cells that are responsible for reconstitution and maintenance of the memory T cell repertoire while sustaining their own pool (Figure 2). T_SCM_ have been described in mice, humans, and non-human primates. T_SCM_ represent 1–4% of total T cells from peripheral blood [61,62], but their presence within tumors or associated draining lymph nodes can average 10–20% in lung, renal, or breast cancer [63,64]. Their presence was also detected in primary human melanoma [65]. T_SCM_ are characterized by the phenotype CD45RA^+^CCR7^+^CD62L^+^ typically associated to naïve cells, but they are notably distinguished by their cell surface expression of CD95 and CD122 (also called IL2-Rβ) memory markers, their high amount of stem cell antigen-1 (Sca-1), and TCF1 transcription factor (encoded by Tcf7) expression [66,67,68,69,70,71] (Figure 2). In infections and cancers, T_SCM_ express ICPs such as PD-1 or LAG3 but at lower levels than in dysfunctional T cells [65,71,72]. They also possess antitumor activity as well as increased survival and proliferative capacity over T_CM_ and T_EM._ This endows T_SCM_ with a superior protective efficacy against tumor growth in mouse models [66,67] (Figure 2). Besides, in response to antigen loss, specific T_SCM_ do not undergo attrition such as T_CM_ or T_EM_, but their populations are maintained while remaining quiescent [73,74,75]. Thus, T_SCM_ ensure the preservation of the T cell repertoire diversity following the occurrence of tumor antigen silencing, which is a natural phenomenon of the tumor escape mechanism. This also indicates that they represent the memory footprint of the history of the host’s antitumor T cell immunity. In the study by Biasco et al., the presence of engineered T_SCM_ with conserved renewal potential was detected up to 12 years after their infusion into bone-marrow transplanted patients [76]. Since some human cancers can still be detected many years after current treatments, these findings highlight the potential of T_SCM_ as persistent cells that could durably preclude cancer recurrence and rationalize the development of therapeutic strategies stimulating these cells.

**Figure 3:**
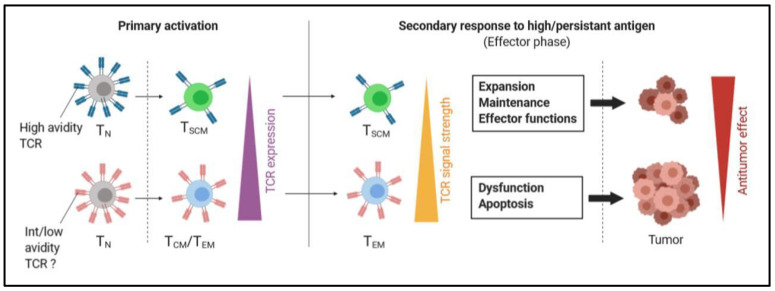
T_SCM_ and T_CM_/T_EM_ express distinct T-cell receptor (TCR) clonotypes that are differently regulated to respond to high/persistent antigen. T_SCM_ are preferentially differentiated from naïve T cells with high avidity TCR clonotypes, and their primary activation drives a drastic downregulation of their TCR. Compared to conventional memory T cells, this results in the triggering of low TCR signals, which allows T_SCM_ to maintain an efficient antitumor response in the presence of persistent antigens during the effector phase. T_N_, naïve T cell; T_SCM_, stem-cell like memory T cells; T_CM_: central memory T cell; T_EM_, effector memory T cell.

Recent studies indicate that vaccines may be an effective way for the stimulation and expansion of specific T_SCM_. Notably, the work of Siddiqui et al. conducted in mice highlighted the critical impact of pre-existing T_SCM_ in the therapeutic efficacy of vaccines. In their experiment, GP133-specific Tcf7^+^ or Tcf7^-^ CD8 T cells were transferred into mice before B16-GP133 melanoma engraftment and vaccine administration. Mice transferred with Tcf7^-^ CD8 T cells (which do not contain T_SCM_) had a significant decrease of overall survival, indicating that the absence of pre-existing specific TCF1^+^ T cells negatively affected the vaccine efficacy [65], which was probably by impairing the development of memory T cell precursors [77]. Furthermore, Wu et al. demonstrated that a vaccine able to stimulate specific T_SCM_ has greater antitumor efficacy than those that exclusively give rise to T_CM_ or T_EM_. Importantly, the authors also established that specific CD8^+^ T_SCM_ are preferentially generated from cells expressing high avidity T-cell receptors (TCRs); however, these are quickly downregulated after stimulation, lowering the sensitivity of CD8^+^ T_SCM_ to antigen [75]. As a result of this weak TCR signaling during the effector phase, CD8^+^ T_SCM_ maintain their effector functions while being protected against an overactivation that may drive their dysfunction and apoptosis when their reencounter persistent antigen. These data are in line with other reports stating that weak stimulatory signals promote the induction of T_SCM_ expressing high-avidity TCRs [67,69] (Figure 3). It also suggests that some T cell clonotypes are likely more susceptible to differentiate into T_SCM_ than others. Thus, identifying such tumor-specific TCR could guide the development of T_SCM_-based immunotherapies through specific vaccines or the transfer of genetically engineered T cells.

In metastatic melanoma, a progressive enrichment of tumor-specific CD45RA^+^CCR7^+^CD95^+^CD8^+^ T_SCM_ with high-avidity TCR was observed in the blood of patients having received monthly doses of native peptide-based vaccine [78], suggesting the transferability of the concept in humans (Figure 3). Strategies involving the use of CAR T cells enriched with T_SCM_ have also been recently developed [79,80,81]. Such chimeric antigen receptor (CAR) T cells are currently tested in clinical trials in myeloma (NCT03288493) and multiple solid cancers (NCT02107963).

#### 2.2.2. Harnessing T_RM_ Cells in Cancer

Recently, a novel population of memory T cells usually characterized by core phenotypic markers CD45RO^+^CD69^+^CD103^+/-^ [82,83] and the constitutive expression of checkpoint receptors (ICPs) [84,85,86,87] was defined as tissue-resident memory T cells (T_RM_) (Figure 2). Originally identified in mouse infection models, T_RM_ play an important role in immune homeostasis [88] and tumor immunosurveillance [89]. Contrary to T_CM_ and T_EM_, T_RM_ usually do not re-circulate through blood but are confined within tissues surrounding infected sites such as lung, skin, liver, and intestine, allowing them to be quickly available to control nearby secondary infections or disease spreading [90,91,92,93,94,95,96]. Consequently, this ‘ready-to-go’ subset displays effector functions more promptly compared to other memory T cells, and their cross-talk with resident dendritic cells also contributes to amplify the antitumor activity of other immune cells [97,98]. Importantly, phenotypic similarities and differences were observed between CD8 and CD4 T_RM_, the latter being also more heterogeneous and exhibiting greater clonal diversity [86,99]. Although less studied in a context of cancer, evidence in infection models indicate that CD4 T_RM_ play a critical role in orchestrating the local recall response [100,101]. In mouse and human tumors, tumor-infiltrating CD8 T_RM_ (referred as CD8 TIL_RM_) can represent up to 30% of CD8 T cells [102,103]. The presence of tumor-infiltrating CD103^+^ T_RM_ is associated with improved outcome or recurrence-free survival in many solid human cancers including melanoma, lung, ovarian, cervical, bladder, urothelial, and breast cancer [104].

Like in infection models [105,106], T_RM_ were efficiently generated in animal models after cancer vaccines promoting T_H_1-immune responses and their frequency of induction were dependent on the selected vaccination route. In mice, both intranasal and intramuscular injections of a papilloma virus-based vaccine efficiently controlled the growth of orthotopic head and neck and lung cancer. However, only the intranasal route generated high levels of mucosal CD8^+^ T_RM_ responsible for the prolonged antitumor efficacy of the vaccine. T_RM_ efficiently protected mice against a tumor re-challenge, even when the recruitment of effector cells was blocked [107,108]. In a melanoma mouse tumor model, intradermal but not intraperitoneal cancer vaccine elicited a protective immune response dependent on skin CD8^+^ T_RM_ [109]. The cervicovaginal vaccine was also able to control a genital tumor through a major induction of CD8^+^CD103^+^ T_RM_ tumor-infiltrating lymphocytes (TILs) [110]. Thus, the mucosal or epithelial route has to be privileged for the induction of T_RM_ by vaccines.

Interestingly, the nature of dendritic cells (DCs) that prime T cells seems to contribute to the optimal generation of T_RM_. CD103^+^DC, CD8a^+^ DC [111], or CD301b^+^ DC [112] fostered the differentiation of CD8 T cells toward a T_RM_ phenotype. In humans, lung-resident CD1c^+^ DC achieve similar results through their expression of membrane-bound TGF-β1 (tumor growth factor beta-1) [113]. Considering these observations, it may be interesting to develop a specific DC-based vaccine that could help to orientate the differentiation of specific T cells into T_RM_ more easily. Similarly, the provision of vectorized-IL-15, a cytokine proven to be involved in the formation and maintenance of some category of T_RM_ [114], may be a strategy to use in combination with vaccines. In melanoma, high levels of intratumor IL-15 mRNA were associated with an increased presence of CD8 TIL_RM_ and better survival [102]. Another intriguing feature of T_RM_ is their putative resistance to conventional radiation. A recent study conducted in a model of mouse colorectal cancer reported that TILs resistant to local radiation therapy express a genetic profile similar to T_RM_. The depletion of TGFβ—a key cytokine involved in T_RM_ generation [82,83]—tends to abrogate this resistance [115]. This finding reinforces the importance of T_RM_ in cancer and would make radiation compatible with therapeutic strategies promoting T_RM_ effector functions.

#### 2.2.3. How can the Generation of T_RM_ and T_SCM_ Foster the Efficacy of ICPi?

In metastatic melanoma, PD-1 blockade enhanced the frequency of intratumoral effector memory CD8 T cells, especially in responding patients [116]. Whether this increase was due to the restoration of pre-existing intratumor cells or to the recruitment of novel effectors was elusive. This issue was recently addressed by Yost et al. in a study conducted in human basal/squamous cell carcinoma. The authors found that the clonotypes of tumor-infiltrating CD8^+^ T cells after anti-PD-1 therapy were phenotypically enriched with activation/dysfunction-associated markers including T_H_1-associated cytokines (TNFα, IFNγ), checkpoint receptors (PD-1, TIM-3, LAG-3), and CD103. Importantly, these post-treatment clonotypes mostly differ from the pre-existing CD8 T cell clones [117]. Although the relevance of this observation in other cancers remains to be determined, this finding is of particular interest because, contrary to what was expected, this would suggest that ICPi do not primarily act by reinvigorating CD8 T cells already present in human tumors but rather stimulate the recruitment of novel peripheral CD8 T cells. In this study, approximately 64% (7/11) of anti-PD-1-treated patients had increased the frequency of novel tumor-infiltrating CD8 T clones. These novel clonotypes were particularly found (approximately 50%) among CD8 T clones enriched in TIM-3, LAG-3, and CD103 markers, suggesting a recruitment of bystander and proximal CD8^+^ T_RM_ into the tumor after ICPi therapy. However, how the systemic inhibitors manage to go through the mucosal or epithelial barrier to reach T_RM_ and activate them remains unclear. A small population of tumor-infiltrating CD8 T cells expressing high levels of TCF1-stem cell-like signature was also observed post-treatment.

The contribution of T_RM_ and T_SCM_ to ICPi therapy is supported by the demonstration that PD-1 and/or TIM-3 blockade strongly upregulate their expansion and effector functions [65,102,118], indicating the sensitivity of these cells to this treatment. In human melanoma, the survival of patients treated with anti-PD-1 therapy was found to be positively associated with the pre-existence of CD8 TIL_RM_ which strongly expanded early after the treatment [102]. Compared to total CD8 T cells, the prognostic value of CD8 TIL_RM_ was also more reliable. We and others also documented in lung cancer that peripheral T cells from blood proliferate and secrete IFNγ more intensively after anti-PD-1 blockade [119,120]. Although the exact phenotype of these cells was not determined, a stimulation of blood T_SCM_, which express low levels of PD-1 [72], cannot be excluded.

**Figure 4:**
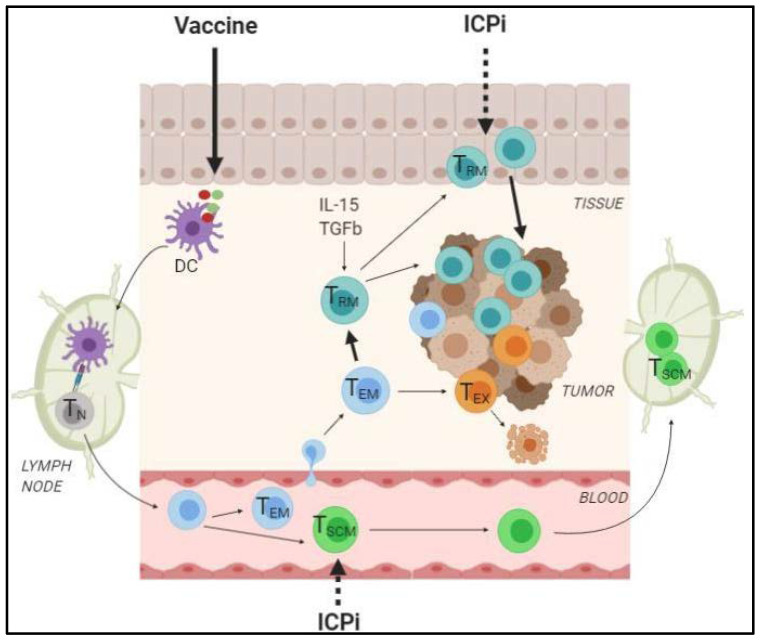
Proposed model of the synergistic antitumor effect of ICPi therapy combined with vaccines. Administration of vaccine by mucosal or skin route can promote the induction and expansion of T_SCM_ and T_RM_ cells, two memory T cell subsets providing long-term protective antitumor immunity through their self-renewal property and immediate effector functions, respectively. In this model, ICPi would primarily act by favoring the recruitment of novel T_RM_ and T_SCM_ clonotypes into the tumor rather than reinvigorating pre-existing dysfunctional T cells. T_N_, naïve T cell; T_SCM_, stem-cell like memory T cells; T_EM_, effector memory T cell; T_RM_, resident-memory T cells; T_EX_, exhausted/dysfunctional T cell; DC, dendritic cell.

In the previous study by Yost and colleagues, the impact of the change of clonality on the clinical response to anti-PD-1 therapy was not investigated, preventing definite conclusions on the benefit of T_RM_ and T_SCM_ recruitment. However, the group of Wilmott identified that a high expression of CD8, CD103, PD-1, and IFNγ-related genes was associated with better outcomes to anti-PD-1 monotherapy in melanoma patients [121]. In addition, in mouse models of melanoma or colorectal cancers, the absence of tumor-infiltrating T_SCM_ significantly reduced the efficacy of ICPi therapies [65,77]. An enrichment in Tcf7 gene was also reported in tumor-infiltrating cells from melanoma patients after anti-PD-1/anti-CTLA-4 therapies [77]. Even though further studies are still required to fully understand the interplay between memory T cells and immunotherapies, all these lines of evidence encourage the use of antitumor vaccines inducing T_RM_ and T_SCM_ in combination with ICPi (Figure 4).

## 3. Immunogenic Therapy and Immune Checkpoint Inhibitors: A Matter of Dose and Timing

### 3.1. ICPi Therapy: A Preventive rather than Curative Care for T Cell Dysfunction?

In clinical settings, patients responding to PD-1/PD-L1 therapy often express high levels of PD-1/PD-L1 in the tumor. However, high levels of tumor PD-1/PD-L1 expression do not always predict clinical response to ICPi therapy. This suggests that immune checkpoint expression alone is not a sufficient predictive biomarker of ICPi response and that the functional status of immune cells expressing these specific molecules is just as important. Indeed, since checkpoint receptors can be expressed on both effector as well as dysfunctional cells [122], an immunological window needs to be considered to rationalize the lack of ICPi efficiency. A progressive strong expression of immune checkpoint receptors and fixed epigenetic alterations eventually lead to a steady-state inhibition of effector T cells, which cannot be further abrogated by ICPi therapy [11,71,123]. In mouse infection models, only PD-1^int^ CD44^+^ but not PD-1^hi^ CD44^+^ CD8 T cells were reinvigorated after PD-1/PD-L1 axis blockade [124]. However, in the context of cancer, most preclinical studies were performed on short-term orthotopic tumors, which may not entirely reflect the dramatic dysfunctional status that human T cells experience after many years of chronic activation in cancer patients. The work of Yost et al. in advanced human cancer showing that anti-PD-1 therapy mainly drives the replacement of pre-existing tumor-infiltrating CD8 T cell rather than reversing their exhausted status supports this statement [117]. Consequently, the existence of a transition point is a strong argument to determine the best timing to disrupt immune checkpoint ligand/receptor interactions before an irreversible dysfunction of pre-existing T cell clones.

The occurrence of some hyperprogressive disease (HPD) has been documented in multiple clinical trials in patients under anti-PD-1/PD-L1 monotherapy, causing the death of the subjects in less than a few weeks [125,126]. The reason of this rapid cancer progression under PD-1/PD-L1 inhibitors is still poorly understood, but it is likely that immune responses play a critical role in HPD. This could possibly occur through overactivation of T cells, leading to inflammation at the tumor site and rapid T cell dysfunction, an increase of PD-1^+^Treg [127], or other immune suppressive cells [128]. Thus, the combination of immunogenic therapy and ICPi has to be carefully defined to benefit from its full potential while limiting the induction of deleterious adverse events.

### 3.2. Concurrent Versus Sequential Combinations: A Balance between Antitumor Efficiency and Immune-Related Side Effects

In most trials, therapies are given concomitantly because this sequence has proven its efficacy in previous studies, but it also allows for a reduction of the period of treatment. However, both in preclinical and clinical settings, the antitumor efficacy of ICPi therapy delivered concurrently with other immunostimulatory treatments raises controversy. In preclinical models, ICPi therapy is often associated to a superior or, at least, identical antitumor effect when given concurrently with immunotherapeutic agents. In our study conducted in mice bearing colorectal tumors (approximately 50 mm^2^), the starting PD-1 blockade simultaneously with Folfox chemotherapy drove rapid and complete tumor regression in 60% of mice [34], whereas delaying or advancing anti-PD-1 therapy by 5 days led to increased tumor relapse (unpublished data). Similar conclusions were made for ICPi therapy combined with other chemotherapies [129] or radiotherapy [37,38,130]. However, Kodumundi et al. recently demonstrated that a sequential administration of anti-PD-1 following a DC-based vaccine was more efficient in controlling breast tumor growth than the concomitant regimen [46]. In a mouse model of B-cell lymphoma, the concomitant injection of anti-PD-1 therapy compromised the antitumor efficiency of anti-4-1BB antibody [131]. Likewise, the study by Messenheimer and colleagues showed in a mouse model of breast cancer that while the concurrent anti-PD-1 injection reduced the antitumor effect of anti-OX40 alone, the delayed administration of anti-PD-1 to anti-OX40 increased therapeutic efficacy [132].

In humans, the survival benefit and acceptable safety of concurrent anti-PD-1 therapy with chemotherapy or chemoradiation over monotherapies have been observed in advanced human cancers such as biliary tract [133] or NSCLC [134,135,136]. However, the simultaneous administration of multiple immunomodulators including radiation to ICPi therapy was also reported to cause fatal adverse events such as pneumonitis or myocarditis, which were likely due to overactivation of the immune system [137,138]. Besides, an augmentation of abscopal cases was reported with patients presenting primary or acquired resistance to ICPi before radiotherapy, although this increase remains minor [139]. In a phase II trial in NSCLC patients, delaying the beginning of anti-CTLA-4 therapy tended to prolong PFS and slightly decreased the occurrence of immune-related adverse effects compared to the concurrent combination [140]. In melanoma, patients receiving chemotherapy after disease progression on the PD-1 blockade had a median OS of 5 years versus only 1.8 years for those receiving either ICPi or chemotherapy alone [141]. The discrepancies between these different combinations may be due to multiple parameters that have to be further investigated to gain additional insights on both the clinical outcome and the adverse effects. Beyond the properties of the combined drugs, the dose and duration of each therapies are factors that affect the antitumor immunity and require refinements for optimal therapeutic efficacy.

### 3.3. Influence of the Dose on the Immunostimulatory Effect of Therapies

To optimize the combination of treatments, it is important that each of them is used at doses that ensure optimal immunological effectiveness. In the clinic, new drugs or treatments are first evaluated in a phase I trial that consists in treating patients with increasing doses of treatment to gather information on safety. In the end, further investigations are based on the maximal dose achieved without side effects. However, does the maximal dose ensure the greatest immunogenicity? Since during phase I clinical trials, the priority is devoted to determining the maximum tolerated dose, few phase I trials addressed the question of the immunogenicity of the dose that is ultimately chosen for further clinical evaluation.

Preclinical studies reported that low-dose vaccines can be at least as effective as high doses. In a mouse melanoma model, Gabri and colleagues explored the antitumor properties of increasing doses of a prophylactic vaccine targeting GM3 ganglioside antigen [142]. Mice received four doses of 120, 240, or 360 μg of vaccine every 14 days; then, B16F10 tumor cells were engrafted 21 days after the last vaccination. All doses were equally capable of protecting mice against tumor growth. In particular, 100% of mice injected with the lowest dose were still alive 35 days after the tumor challenge, indicating that even low doses of the vaccine stimulate optimal and durable antitumor T cell immunity. However, the benefit of the low doses was completely lost when switching to weekly injection instead of every 14 days. This deleterious effect of weekly injections can be explained by an overstimulation during the primary expansion of the specific T cells that eventually led to a premature dysfunction of these cells. The protective effect of low-dose vaccine against viral infections was reported in several clinical trials [143,144,145]. Melief et al. recently evaluated the therapeutic efficacy and toxicity of increasing doses (20, 40, 100 and 300 µg/peptide) of a mix of long-peptide vaccine associated with chemotherapy in a cohort of patients with advanced, metastatic, or recurrent HPV-16^+^ cervical cancer. Severe treatment-related adverse effects such as systemic allergic reaction were more prominent at the 300 µg standard dose. Importantly, there was no difference in vaccine reactivity and overall patient’s survival between the different dose cohorts. However, their study clearly underscores the importance of eliciting a strong antitumor specific T cell immunity, since high response to HPV was associated with OS benefit whatever the dose cohorts [146]. Moreover, the effect of vaccine dosage not only translates on the clinical outcome, but it can also shape the memory phenotype of specific T cells. As previously mentioned, activated T_SCM_ tend to express high avidity but low levels of TCRs, which require high doses and/or persistent antigens to maintain their effector functions [75]. By contrast, high antigen levels drive T_CM_/T_EM_ cell dysfunction. Thus, low-dose vaccine, which may physiologically still be considered as a significant dose, could be enough to ensure the stimulation of these three memory T cell subsets while limiting the generation of terminally dysfunctional T cells that may happen with higher doses and cannot be reinvigorated by ICPi therapy. The study by Gannon et al. showing that T_SCM_ were efficiently generated in melanoma patients treated with a low dose of a native tumor peptide-based vaccine tends to support this idea [78].

Various preclinical studies investigated the strength and durability of the antitumor responses induced during escalating doses of chemotherapy and demonstrated that the maximum tolerated dose was suboptimal [147,148]. In the transgenic adenocarcinoma of the mouse prostate (TRAMP) model, an injection of a low dose (50 mg/kg) of cyclophosphamide ensured the optimal survival of adoptively infused CD8 T cells, while the highest dose (200–400 mg/kg) was highly toxic and induced lymphopenia [149]. Consistent with preclinical data, low doses of cyclophosphamide triggered anti-tumor T cell responses and Treg depletion in advanced human cancers [150,151,152]. Ideally, the dose of chemotherapy should be high enough to ensure the induction of ICD but remain under the threshold inducing severe lymphopenia and immunosuppression. Wu and colleagues proposed the MEDIC model (medium-dose intermittent chemotherapy) to design chemotherapy dose and scheduling to trigger repeated cytotoxic damages to tumor cells, while being compatible with the induction and maintenance of antitumor innate and adaptive immunity [153]. This model could be transposed to other immunogenic therapies to rationally design therapeutic combinations that would increase the efficacy of ICPi.

While we proposed that reactivation of the anti-tumor immune response can be regarded as the 6th R of Radiobiology [154], few studies have investigated whether dose and fractionation of ionizing radiations could modulate systemic antitumor T cell responses [155]. Yovino and colleagues modeled the impact of conventional radiation regimens in patients suffering from high-grade gliomas and showed that after 30 fractions of 2Gy, approximately 99% of circulating T cells received more than 0.5Gy. The mean dose delivered to T cells was 2.2Gy, which is higher than the lethal dose of ionizing radiations required to reduce the surviving fraction of lymphocytes by 50% [156,157]. In mouse models, the multiple administration of low doses of ionizing radiation (3 × 5Gy) induced an increase of MDSCs dependent on CSF1/CSF1R signaling in blood, spleen, and lymph nodes [158]. However, in another study, a single high dose of ionizing radiation resulted in the elimination of MDSCs and triggered efficient priming and the induction of antitumor CD8 T cell responses [159]. Thus, the use of radiotherapy as an adjuvant for immunotherapy requires the challenging identification of the threshold governing the balance between immunosuppressive and proimmunogenic effects. In this respect, Vanpouille-Box and colleagues recently identified the exonuclease Trex1 as an inhibitory factor of radiation-induced immunogenicity whose expression is upregulated by high doses of radiations. The authors showed in mouse mammary carcinoma TS/A that high single or multiple doses of radiations (> 12–18Gy) induced Trex1, which in turn degrades cytoplasmic dsDNA and prevents the activation of the cGAS/STING/IFN I pathway, thus impairing the ability of radiotherapy to stimulate the immune system. The authors demonstrated that the threshold governing Trex1 activation varies according to the histological type of the tumor and needed to be carefully defined to sensitize the tumor to ICPi. The threshold for the induction and activation of Trex1 was indeed 12Gy in the human 4175TR triple negative cancer cell line, while it increased to 18Gy in human MDA-MB-231 metastatic breast cancer cells. Similar differences were also observed in other common mouse tumor models such as mammary 4T1 and MC38 colon cancer [160]. In a recent clinical trial, Formenti and colleagues tested two palliative radiation schemes (3 × 9Gy versus 5 × 6Gy) in combination with anti-CTLA-4 in 39 chemorefractory NSCLC patients. Responses to the therapy were positively correlated with IFN-β secretion and the expansion of circulating T cell clones, and no differences were detected between the two irradiation modalities. However, half of the patients enrolled did not respond to the therapy [57].

With the aim of determining the optimal delivery scheme of radiation with immunotherapy targeting the PD-1/PD-L1 axis and TIGIT (T cell immunoreceptor with Ig and ITIM domains), Grapin and colleagues tested in CT26 tumor-bearing mice 3 irradiation schemes (18 × 2Gy, 3 × 8Gy, and 1×16.4Gy). Interestingly, they confirmed the superior efficacy of the 3 × 8Gy regimen in combination with anti-TIGIT and PD-L1 [155], as similarly reported with anti-CTLA-4 antibodies [160]. A genetic analysis of tumors showed that 3 × 8Gy induced a strong upregulation of genes involved in both cGAS/STING/IFN-I pathways and T cell activation/effector functions. Thus, optimizing the radiation regimen to minimize Trex1 activity and favor IFN-I release should help to obtain better results.

### 3.4. Defining Optimal Duration for ICPi Therapy: When to Stop and Replace?

Another therapeutic parameter that has raised questions since the development of ICPi is the duration of the treatment, especially in patients who achieve complete tumor regression (CR) or those with stable disease (SD) before the completion of ICPi therapy. Is there a benefit in continuing the treatment? Does it minimize the risk of tumor relapse for CR patients? Or in case of partial response (PR) or SD, would that not indicate the necessity of a change of ICP target?

In various clinical trials, patients received anti-PD-1 treatments during a maximum of 2 years [54,55,161,162]. However, such treatment duration merits further consideration, considering that PD-1 inhibitors may impair immune memory formation. For instance, a meta-analysis revealed that in advanced melanoma treatment, discontinuation because of adverse events did not negatively influence treatment outcomes with anti-CTLA-4 and anti-PD-1, suggesting that shorter treatment duration does not necessarily compromise the therapeutic efficacy of ICPi [163]. In a study conducted in advanced melanoma, Jansen and colleagues reported that in comparison to patients showing partial responses (PR) or stable diseases (SD), those with a complete response (CR) seemed to have a lower risk of relapse after ICPi discontinuation [164]. Currently, other trials aim to define the optimal duration of PD-1/PD-L1 inhibitor treatments. The Canadian STOP-GAP study (NCT02821013) is evaluating intermittent versus continuous treatment with anti-PD-1 inhibitors in 614 patients and the OS as primary endpoint. To do so, patients are being randomized in the first 16 weeks of anti-PD-1 therapy to either a standard 2 years of treatment or until the obtention of maximal tumor response followed by retreatment at the time of progression. The DANTE trial (ISRCTN15837212) is a non-inferiority trial in metastatic melanoma with the PFS as primary endpoint. Patients treated with anti-PD-1 therapy who are progression-free at 12 months are randomized to either stop (with re-challenge allowed on progression) or continue standard treatment.

In addition to the potential deleterious effect of ICPi therapy arrest that is under investigation, the absence of complete tumor shrinkage (PR and SD status) in patients under ICPi therapy may also indicate that immunological changes occur in the tumor and counteract the effect of the treatment. This might also happen in some patients with complete response but cancer recurrence after ICPi discontinuation and for whom pursuing or resuming the same therapy may be worthless. In a retrospective study conducted in melanoma patients with complete response to anti-PD-1 therapy, the probability of being alive after 3 years was 72%. However, most patients who relapsed were thereafter unresponsive to retreatment. Of note, no association was found between anti-PD-1 duration and tumor relapse [165]. Similar to other conventional therapies, an acquired resistance to PD-1/PD-L1 inhibitors can arise including an upregulation of other inhibitory molecules such as TIM-3 [166,167,168,169], thus rendering the last ICPi-based therapy no more sufficient. This clearly emphasizes the importance of ensuring a continuous monitoring of patients’ antitumor T cell immunity and associated immune regulators during the treatment to anticipate when the current ICPi needs to be stopped and possibly replaced by another one.

## 4. Conclusions and Perspectives

**Figure 5:**
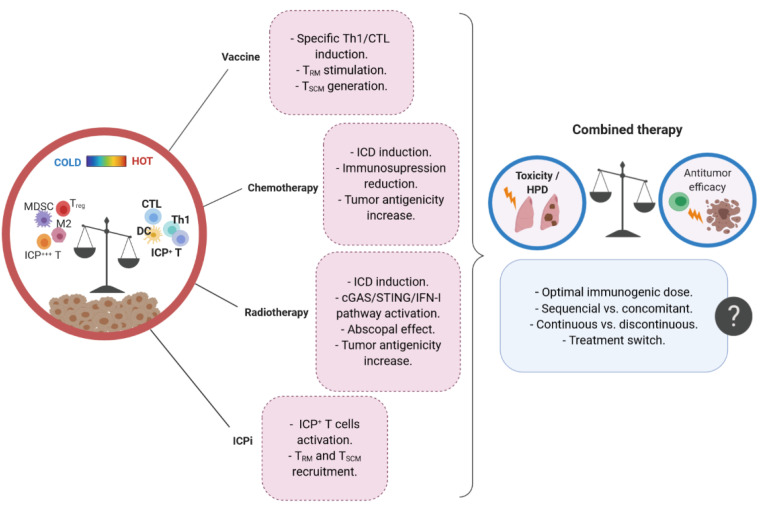
Parameters influencing the antitumor efficacy and toxicity of combined immunogenic therapies. Vaccines, immunogenic chemotherapy/radiotherapy, and immune checkpoint inhibitors (ICPi) act by promoting the activation of the immune system, converting ‘cold’ tumor in ‘hot’ tumors. However, treatment combination warrants special attention about the sequence, dose, and duration of each therapy to ensure durable antitumor response while avoiding deleterious immune-associated adverse events. MDSC, myeloid-derived suppressive cells; CTL, cytotoxic T cell; M2, type-2 macrophage; ICD, immunogenic tumor cell death; T_RM_, tissue-resident memory T cell; T_SCM_, stem-cell like memory T cell; HPD, hyperprogressive disease.

The use of immunogenic therapies to overcome resistance to ICPi is based on evidence that the accumulation of ICP^+^ T cells with effector functions within the tumor before ICPi therapy is associated with a good prognosis. To design such therapeutic strategies, the combined treatments must be wisely chosen to elicit appropriate immunostimulatory signals and regulate the balance between pro- and antitumor immunity. Combining ICPi to therapies depleting immunosuppressive cells while stimulating the activation and recruitment of T cell immunity is a strategy that has proven its efficiency in various preclinical studies. More recently, attention was drawn toward the elicitation of tumor-specific ICP^+^ T_SCM_ and T_RM_ cells due to their respective properties of self-renewal and local effectors. In human cancers, the presence of T_SCM_ or T_RM_ cells is associated with better survival, and two studies reported that these populations seem to be particularly mobilized during anti-PD-1 therapy [102,117]. The rationale of combining immunogenic therapies to ICPi led to several clinical trials testing different associations in multiple cancers [170,171,172,173,174]. While clinical studies tend to confirm, at least in some cancer types, the benefit that such combinations bring to patients, the results clearly indicate that improvements are still required to fully exploit the potential of combined therapies while limiting their immune-related toxicity. A better understanding of the kinetics of anticancer immune responses and their associated regulators is critical to optimize the sequence, dose, and duration of the combined treatments (Figure 5). Thus, it is essential to determine the immune and molecular changes occurring in patients throughout combined therapies to get a comprehensive understanding of their efficacy or predict instead a possible relapse, leading to a change in treatment. Results from immunomonitoring studies will be instrumental to design and refine therapeutic strategies with durable efficacy.
